# Perspective and quality of life in amyotrophic lateral sclerosis patients undergoing percutaneous endoscopic gastrostomy

**DOI:** 10.3389/fnut.2026.1756642

**Published:** 2026-06-19

**Authors:** Nilo Riva, Enrica Finotto, Paride Schito, Giorgia Donzelli, Tommaso Russo, Teuta Domi, Laura Pozzi, Andrea Tettamanti, Elisa Riboldi, Ignazio Diego Lopez, Angelo Quattrini, George Cremona, Mauro Comola, Massimo Filippi

**Affiliations:** 1 Neurology and Neuro-rehabilitation Unit, Division of Neuroscience, IRCCS San Raffaele, Milan, Italy; 2 Experimental Neuropathology Unit, Division of Neuroscience, Institute of Experimental Neurology (INSPE), San Raffaele Scientific Institute, Milan, Italy; 3 Department of Rehabilitation and Functional Recovery, IRCCS San Raffaele Scientific Institute, Milan, Italy; 4 Unit of Respiratory Medicine, IRCCS San Raffaele Scientific Institute, Milan, Italy; 5 Vita-Salute San Raffaele University, Milan, Italy

**Keywords:** cachexia, dysphagia, motor neuron disease, nutrition, quality of life, rehabilitation, respiratory failure, survival

## Abstract

**Introduction:**

Percutaneous endoscopic gastrostomy (PEG) is commonly used to manage dysphagia and nutritional failure, which are among the most frequent and severe complications of amyotrophic lateral sclerosis (ALS). While several studies assessed PEG indications, outcomes, and prognostic factors, there is no evidence regarding ALS patients’ perspectives and health-related quality of life (HRQoL) associated with PEG.

**Methods:**

This study included 48 consecutive ALS patients. At the 1-month follow-up after PEG, patients and their caregivers completed a PEG satisfaction questionnaire regarding their decision to proceed with the PEG-tube placement. HRQoL was assessed using the Gastrointestinal Quality of Life Index (GIQLI) and the Short Form-36 (SF-36).

**Results:**

In total, 77.1% of patients and 88.9% of caregivers confirmed that they would prefer to have a PEG tube placed again if required (*p* > 0.001); 93.8% of patients felt that PEG made feeding easier, exerting a positive effect on overall wellbeing (83.3%) and increasing survival rates (93.8%) (*p* > 0.001); 54.2% felt that PEG was cosmetically acceptable. Consistent positive rates were reported by caregivers. The GIQLI digestion subscale values significantly improved from baseline (28.3; SD = 6.6) to discharge (30.97, SD = 5.84) and were maintained at 1-month follow-up (30.21, SD = 6.7; *p* = 0.014). Conversely, in follow-up assessments, we observed a significant reduction in the SF-36 physical component summary (PCS) subscale (baseline = 33.3; 1-month follow-up = 28.61; *p* = 0.032), which was accompanied by a significant worsening in the GIQLI physical dimension subscale (baseline = 9.63; 1-month follow-up = 7.38; *p* = 0.044).

**Discussion:**

This study provides preliminary evidence that ALS patients have a positive perspective on PEG positioning, which may also have a beneficial effect on HRQoL related to gastrointestinal function.

## Introduction

Dysphagia is one of the most frequent complications of amyotrophic lateral sclerosis (ALS), which is a relentless progressive disease defined by the degeneration of upper and lower motor neurons in the brain and the spinal cord (1). While bulbar ALS, one of the most severe disease variants, accounts for approximately 25–30% of cases, the majority of ALS patients eventually suffer from speech and progressive swallowing difficulties (1, 2), leading to consequent weight loss, dehydration, choking, a substantial increase in the risk of aspiration pneumonia, and, ultimately, nutritional failure (1, 3–6). Therefore, monitoring bulbar symptoms is one of the cornerstones of the multidisciplinary follow-up program for ALS patients. The initial management of dysphagia involves counseling from dietitians and speech therapists. This may include modifying the consistency and composition of food, providing food supplements, and implementing specific postural maneuvers (3, 7–9). As dysphagia worsens, a decision should be made on whether enteral nutrition is an acceptable intervention as an alternative or supplemental option for oral nutrition (4–6, 9). Although various techniques can be used for gastrostomy tube (G-tube) insertion, percutaneous endoscopic gastrostomy (PEG) has traditionally been the method of choice for patients with ALS (7, 10–14). While many questions remain regarding the individual complexity of each patient, several studies have previously assessed indications (11, 15), outcomes (7, 10, 12, 15–17), and prognostic factors related to PEG positioning in ALS patients (13, 16, 18–21), leading to a general consensus on the need for early discussions about the insertion of a feeding tube to optimize safety and benefits (4, 5, 7, 9). However, the evidence supporting PEG’s impact on survival remains limited (6, 22).

Despite the findings of these studies, there is no evidence regarding patients’ perspectives and health-related quality of life (HRQoL) in people with ALS undergoing PEG, which is recognized as a major gap in knowledge in this field (5, 6). Indeed, ALS has a profound impact on HRQoL for both patients and the whole family unit, as a consequence of the relentless loss of independence, co-existence of cognitive and behavioral deficits, reduced ability to communicate, eat, swallow, cough, and breathe. This deterioration can ultimately lead to nutritional and respiratory failure (23). In the absence of a cure, the primary focus of management is on symptom control, with greater benefits when treated in specialized, multidisciplinary ALS clinics (1, 24, 25). Hence, it is crucial to assess patients’ reported outcomes and the impact of HRQoL preservation (23).

Against this background, the aim of this study is to explore patients’ and caregivers’ perspectives and HRQoL in a cohort of ALS patients undergoing PEG.

## Materials and methods

### Patient selection and evaluation

This study included a cohort of 48 consecutive ALS patients who underwent PEG positioning after providing informed consent and were willing to participate in this HRQoL study, which was approved by the Local Ethic Committee. The patients were hospitalized in the Neurorehabilitation Ward at the San Raffaele Hospital in Milan between October 2007 and July 2020. Out of a total of 131 patients for whom PEG was considered, 40 patients (31.8%) were excluded from the analysis due to the absence of HRQoL test data. Additionally, 43 patients (32.8%) who did not undergo the procedure because they either refused it or it was deemed contraindicated based on factors such as high-risk/low-benefit, being in a terminal stage, or having comorbidities (Supplementary Figure S1) (4, 5). ALS diagnosis was based on the revised El Escorial criteria. Neurological evaluation included demographic information, the site of onset, disease duration at the time of PEG, diagnostic delay, and the MRC sum score (range: 0–120) (26). Disability was assessed using the revised ALS functional rating scale (ALSFRS-R), and the bulbar and respiratory subscores were separately derived. The rate of disease progression was calculated by using the following formula: (48 – ALSFRS-R score)/disease duration (27, 28). Respiratory function was assessed using forced vital capacity (FVC) and arterial blood gas (ABG) analyses. Weight loss in the previous 5 months was recorded, while nutritional status was assessed using body mass index (BMI) and routine blood tests (29). At admission, dysphagia was systematically assessed in a multidisciplinary setting by both a speech therapist and an otolaryngologist using either fiberoptic endoscopic evaluation of swallowing (FEES) or a videofluoroscopic study. Recommendations for enteral feeding with PEG were based on specific criteria, including severe dysphagia (ALSFRS-R swallowing subscore ≤2), significant unintentional weight loss (>10% of premorbid weight or >5% in the previous 5 months), doubled meal times, and anamnestic repeated aspiration (5, 15). Respiratory function was assessed in all PEG candidates before the procedure. At admission, 8/48 patients (16.7%) were already receiving nocturnal non-invasive mechanical ventilation (NIMV), while 9/48 (18.7%) were initiated on NIMV before PEG (9). NIMV was maintained during the procedure in three cases using a laryngeal mask (30), and in all cases, it was reapplied immediately after the procedure using the patient’s own oro–nasal interface. Tracheostomy-free survival was also recorded.

### Patient perspectives and HRQoL

PEG Satisfaction Questionnaire: At 1-month follow-up, patients and their caregivers were given a PEG satisfaction questionnaire regarding the decision to repeat PEG-tube placement if necessary (31). It included the assessment of both the advantages of the PEG tube, such as ease in feeding, cosmetic acceptability, increased survival duration, and its impact on physical wellbeing, as well as the possible disadvantages, including increased time consumption in feeding, dependency on others for feeding, and any cost issues related to care (31).

HRQoL was assessed using two different questionnaires: the Gastrointestinal Quality of Life Index (GIQLI) (32, 33) and the Short Form-36 (SF-36) (34), administered upon admission, at discharge, and during a 1-month follow-up. The GIQLI is a reliable 36-item self-report measure specifically designed for individuals with different gastrointestinal disorders, assessing HRQoL related to esophageal/gastrointestinal dysphagia (32, 33). Items are summed to produce a total score (range: 0–144, with higher scores denoting better QoL) (32). Four subscales were defined: physical dimension (items 15–21), gastrointestinal digestion (items 1–6, 27, 28, 32, 35), gastrointestinal defecation (items 7, 26, 30, 31, 34, 36), and mental wellbeing (items 10–14) (32, 35). The SF-36 consists of 36 items, which are aggregated to calculate scores for 8 sub-scales, as well as to generate the physical component summary (PCS) score and the mental component summary (MCS) score (range: 0–100, with higher scores denoting better QoL) (23, 36).

### Statistical analysis

Descriptive statistics were used to summarize the variables of interest. Comparison between groups were performed using Fisher’s exact test, *t*-test, Mann–Whitney U test, analysis of variance, or Kruskal–Wallis test, as appropriate for the type and distribution of the variables. The Friedman test for repeated measures, followed by a Wilcoxon post-hoc test, was applied to assess changes in HRQoL over time. Confidence intervals were estimated by bootstrapping (1,000 resamples). To address potential selection bias, sensitivity analyses were performed using multiple imputation (MI) based on baseline predictors, last observation carried forward (LOCF), and inverse probability weighting (IPW); E-values were used to assess the robustness of the findings against unmeasured confounding (Results in the Supplementary material). Survival was estimated using Kaplan–Meier curves and the log-rank test. Data were analyzed using SPSS 22.0 software (Technologies, Inc., Chicago, IL, USA). Significance was set at a *p*-value of < 0.05.

## Results

HRQoL questionnaire responses were obtained from 48 ALS patients (Table 1). The mean age at onset was 65 years, while the average disease duration at the time of PEG was 36.4 months. Up to 48% of patients presented with bulbar onset, and the mean bulbar score was markedly reduced (mean value: 5.7; SD: 2.76). While the mean BMI was 21.56 (SD: 4.01), 18.8% of patients presented with a BMI of <18.5. At the time of PEG positioning, 47.9% of patients were already receiving NIMV. While no short-term mortality was observed in the cohort, postoperative pain was reported in 51.4% of patients. The overall population of patients who underwent PEG positioning showed, compared with non-PEG recipients, a higher percentage of bulbar onset (28.6% vs. 48.4%: *p* < 0.05), lower ALS-FRS-R progression rates (1.6 vs. 1.2: *p* < 0.05), lower mean ALS-FRS-R-bulbar scores (6.8 vs. 5.2: *p* < 0.05), and higher mean ALS-FRS-R-respiratory scores (7.7 vs. 9.6: *p* < 0.05). The percentage of NIMV users was 66.7% in the PEG group compared to 48.4% in the non-PEG group (*p* < 0.05) (Supplementary Table S1). Within PEG recipients, patients included in this perspective study presented lower rates of dementia compared with those for whom QoL tests were not available, (14.6% vs. 35.6%: *p* < 0.05), and higher mean FVC values (65.8% vs. 50.0%: *p* < 0.05) (Supplementary Table S2). The subgroup of PEG recipients included in this study (Supplementary Table S2) showed significantly longer overall and post-PEG survival compared to both patients who did not receive PEG (*p* = 0.013 and *p* > 0.001) and PEG recipients whose tests were not available (*p* = 0.040 and *p* < 0.001) (Supplementary Tables S3, S4; Supplementary Figure S2).

**Table 1:** Baseline demographic and clinical data of study patients.

Variable	Values N: 48–n (%); m [SD]
Age at onset (years)	65.05 [11.06]
Gender (M:F)	18:30 (37.5%:62.5%)
Diagnostic delay (months)	16.54 [27.52]
Site of onset (bulbar:spinal)	23:25 (47.9%:52.1%)
Disease duration at the time of PEG (months)	36.41 [45.25]
El-Escorial: definite; possible; probable laboratory supported; probable	24 (50%): 14 (29.2%): 4 (8.3%): 6 (12.5%)
ALSFRS-R-Total	25.94 [8.92]
ALSFRS-R bulbar score	5.67 [2.76]
ALSFRS-R respiratory score	9.56 [2.38]
ALSFRS-R progression rate	1.12 [0.86]
MRC sum score	83.07 [29.13]
Dementia (yes:no)	7:41 (14.6%:85.4%)
BMI (kg/m^2^); n < 18.5 (%)	21.56 [4.01]; 9 (18.8%)
Weight loss (previous 3–6 months; kg; %)	5.52 [5.33]: 8.42% [7.61] (n:21)
FVC (% pred)	65.79 [22.43]§ (n:39)
NIMV (yes:no)	25:23 (52.1%:47.9%)

Values are presented as the number of subjects (%) and mean values [SD]; SD: standard deviation. Data were available for the all-study group (N: 48 patients). if not otherwise specified. @: n: 31; §: n:39. BMR: basic metabolic rate; H-B: Herris Benedict. # Hyper-hypometabolism is defined as measured resting energy expenditure (MREE) at indirect calorimetry / HB-predicted REE > 110% or < 110%, respectively.

The analysis of patients’ perspectives, obtained 1 month after PEG placement, showed that the majority of ALS patients (77.1%) and their caregivers (88.9%) would choose to have a PEG tube placed again if required (*p* > 0.001 for both) (Table 2). Additionally, most patients (93.8%) and all caregivers (100%) felt that PEG made feeding easier, exerting a positive effect on overall wellbeing (patients: 83.3%; caregivers: 82.2%) and increasing survival (patients: 93.8%; caregivers: 95.5%; *p* > 0.001). The majority of patients felt that PEG was cosmetically acceptable (patients: 54.2%; caregivers: 68.8%; *p* > 0.001). Concerning potential disadvantages, a notable proportion of patients (62.5%) and caregivers (68%) felt that PEG increased the time required for feeding. Additionally, many patients (72.9%) and a notable number of caregivers (64.4%) felt that PEG led to greater dependency on others for feeding. However, the majority of both patients (45.8%) and caregivers (55.5%) did not report an increase in cost of care.

**Table 2:** Patients/caregivers’ perspective regarding PEG-tube placement.

Question	Yes	No	Indecisive	*p*-value
Patients (N: 48)				
Would you like to have PEG tube again if required?	37 (77.1%)	8 (16.7%)	3 (6.3%)	0.000
Ease in feeding	45 (93.8%)	1 (2.1%)	2 (4.2%)	0.000
Cosmetically acceptable	26 (54.2%)	14 (29.2%)	8 (16.7%)	0.004
PEG tube increases the survival	45 (93.8%)	1 (2.1%)	2 (4.2%)	0.000
Positive effect on overall wellbeing	40 (83.3%)	3 (6.3%)	5 (10.4%)	0.000
Increase time consumption due to feeding	30 (62.5%)	11 (22.9%)	7 (14.6%)	0.000
Dependent on the others for feeding	35 (72.9%)	11 (22.9%)	2 (4.2%)	0.000
Increase in the cost of care	29 (39.6%)	22 (45.8%)	7 (14.6%)	0.022
Caregivers (N: 45)				
Would you like to have a PEG tube again if required?	40 (88.9%)	0	5 (11.1%)	0.000
Ease in feeding	45 (100%)	0	0	0.000
Cosmetically acceptable	31 (68.8%)	6 (13.3%)	8 (17.9%)	0.000
PEG tube increases the survival	43 (95.5%)	0	2 (4.5%)	0.000
Positive effect on overall wellbeing	37 (82.2%)	1 (2.2%)	7 (15.5%)	0.000
Increase time consumption due to feeding	31 (68%)	7 (15.5%)	7 (15.5%)	0.000
Dependent on the others for feeding	29 (64.4%)	13 (28.8%)	3 (6.8%)	0.000
Increase in the cost of care	12 (26.7%)	25 (55.5%)	8 (17.8%)	0.005

Values are shown as the number of subjects (%). To compare categorical variables. A two-tailed *p*-value Fisher’s exact test of k-proportions was applied.

At baseline, a marked reduction in the SF-36 HRQoL scores was observed compared to the normative values for Italy, as expected (Table 3) (34). At follow-up, a significant reduction in the PCS SF-36 summary subscale scores was observed (baseline: 33.3; 1-month follow-up: 28.61; *p*: 0.032), paralleled by a significant worsening in the GIQLI physical dimension subscale scores (baseline: 9.63; 1-month follow-up: 7.38; *p*: 0.044). These findings are consistent with the expected relentless ALS disability progression. The SF-36 MCS summary scale and the GIQLI mental wellbeing subscale scores did not change over time. Interestingly, when analyzing the GIQLI subscale that specifically assesses gastrointestinal digestion, a significant improvement was noted from the baseline mean values (28.3; SD: 6.6) to discharge values (30.97, SD: 5.84), maintained at 1-month follow-up (30.21, SD: 6.7; *p*: 0.014). No significant changes were observed in the other GIQLI subscales.

**Table 3:** Values at baseline, discharge, and at FU (Friedman test).

Parameters	T0–Baseline (pre-PEG)	T1–Discharge	T2–1 month FU	*P*
*SF-36*				
Physical component summary (pcs)	33.28 (10.86)	30.37 (8.27)	28.61 (8.70)	**0.034**
Mean change (95% CI)		*T1-T0: −2.39 (−5.04/0.34)*	T2-T0: −4.21* (−1.61 / -6.97)	*T2-T1: −1.48 (−4.60/1.84)*
Mental component summary (mcs)	35.21 (12.55)	38.18 (13.05)	36.83 (12.70)	0.605
*GIQLI*				
Physical wellbeing subscale	17.17 (6.88)	16.31 (6.43)	13.83 (5.32)	0.404
Physical dimension	10.48 (6.69)	9.17 (5.81)	7.10 (4.60)	**0.045**
Mean change (95% CI)		*T1-T0: −1.11 (−3.05/0.58)*	T2-T0: −2.67* (−4.70/−0.65)	*T2-T1: −0.87 (−3.17/1.25)*
Mental wellbeing subscale	8.97 (4.23)	8.83 (4.08)	8.97 (4.10)	0.717
Gastrointestinal digestion subscale	28.38 (6.60)	30.97 (5.84)	30.21 (6.67)	**0.014**
Mean change (95% CI)		T1-T0: 3.28* (1.47/5.02)	*T2-T0: 1.80 (−0.05/3.73)*	*T2-T1: −1.87 (−3.50/−0.27)*
Gastrointestinal defaecation subscale	18.72 (3.73)	18.97 (3.27)	17.28 (4.54)	0.177
Total subscales	83.72 (17.07)	84.86 (17.02)	79.10 (19.25)	0.072

Values are presented as number of subjects (%) and mean values (SD); sample size: SF-36: N: 23; GIQLI: N: 29. Significant differences at the Friedman test are shown in bold. Post hoc differences are shown in italics. *Post-hoc* analysis shows mean changes over time with bootstrapped confidence intervals (1.000 resamples). *p < 0.05.

The results of the sensitivity and IPW analyses were generally consistent with our primary findings, confirming a beneficial effect on psychological subscales despite an overall decline in patients’ physical functioning (Supplementary Tables S5–S14). E-values were used to estimate the minimum strength of association that an unmeasured confounder would need to nullify findings, mitigating potential selection bias due to the limited sample size (Supplementary Tables S15, S16).

## Discussion

While PEG is often used to manage dysphagia and nutritional failure in ALS patients, few studies have examined how they experience this intervention or how it may affect their HRQoL. In this study, the vast majority of patients confirmed that they would choose to have a PEG tube placed again if required, showing an overall positive perspective toward PEG after the intervention. Additionally, by utilizing the GIQLI (32, 33), the study detected an improvement in HRQoL associated with gastrointestinal symptoms, even during the terminal stages of the disease.

PEG is a common intervention for dysphagia and nutritional failure in ALS patients, even though the evidence supporting its benefits regarding survival and quality of life remains limited (5, 8, 9). The adoption of PEG varies substantially across regions, reflecting sociocultural influences on decision-making (37, 38). In the absence of validated prognostic algorithms, early discussion of inserting a feeding tube is currently recommended, suggesting PEG positioning before FVC decreases below 50% of the predicted level (4, 7, 9, 15). Against this background, the results of this study show that the majority of ALS patients would choose to have a PEG tube placed again if required. Additionally, the majority of patients felt that PEG was cosmetically acceptable and made feeding easier, exerting a positive effect on overall wellbeing and survival, despite potential disadvantages such as an increased time consumption due to feeding and a greater dependence on others for feeding.

The decision to obtain a PEG is often challenging for patients and caregivers, as reflected by recent observational studies indicating that among individuals with ALS, the mean delay from the clinical indication for PEG to actual placement can be as long as 8 months, reflecting substantial variability driven by patient clinical factors and decision-making (39, 40). Patient concerns include procedural fears, activity limitations, body image concerns, and concerns about life prolongation (41). Despite these potential concerns and misunderstandings, a high level of satisfaction after PEG placement (77.1%) was observed. This finding is consistent with a recent study of ALS patients that reported a satisfaction rate of 93% (41), and is higher compared to a previous study involving patients with non-ALS neurological disorders (60%) (31). However, it is acknowledged that in this study and previous studies, these findings may also be influenced by selection bias, as this factor was not controlled for and we were unable to obtain information from the entire patient population. Up to 94% of patients perceived that PEG was useful in increasing ease in feeding, which is consistent with the recognized potential benefits of PEG and is in line with a recent cohort study showing that 75.6% of ALS patients reported that gastrostomy feeding was easier than they had expected post-procedure (5, 9, 41). Notably, while the satisfaction questionnaires suggest that patients believe PEG prolongs survival (94%), this claim is not definitively supported by current evidence (5, 7–17, 19–22, 25, 42–44). This discrepancy may reflect expectation bias among patients undergoing PEG. Additionally, we were unable to obtain data from the entire cohort, and patients who completed questionnaires had longer survival rates compared to those for whom data were unavailable. Conversely, a previous study found that 26% of patients expressed concern that a feeding tube could extend life (41). Unfortunately, this study was unable to obtain comparable data from non-PEG recipients in our cohort. Despite the relative invisibility of PEG tubes under usual attire, one of the potential barriers affecting patient decisions regarding gastrostomy tube placement is the impact on body integrity and image, which is reported in up to 30% of patients prior to PEG placement (41, 45). Even though direct comparisons were not possible, 54.2% of patients considered PEG cosmetically acceptable post-intervention, while 45.8% found it unacceptable or remained indecisive. This finding highlights an underestimated issue for ALS patients concerning body image that persists after placement, despite the high overall satisfaction rates (45). Notably, caregivers’ perspectives were consistent with responses provided by the patients, with comparable, even more frequent positive response rates, in line with a previous study (31).

Despite the perceived benefits, one of the potential drawbacks of PEG positioning is the increased time required for feeding and the greater dependency on others for feeding, consistently reported by approximately 70% of both patients and caregivers. This apparent discrepancy may be partially explained by the complex relationship between feeding efficiency and the overall burden placed on caregivers after PEG placement. Recent observational studies demonstrate that both patients and caregivers initially experience decreased stress related to feeding after PEG placement (41). However, the ProGas prospective cohort study found that overall caregiving strain increased significantly at 3-month post-gastrostomy (14). This suggests that while PEG eliminates the stress of prolonged oral feeding attempts, the total caregiving burden rises due to the technical demands of PEG management and the underlying progression of ALS. Although the National Health System in Italy covers the costs of feeding formula, tubing, and ancillary supplies, only 45.8% of patients and 55.5% of caregivers reported no perceived increase in the cost of care following PEG placement. The remaining respondents were either indecisive or reported an increase in care-related costs. These results are consistent with a previous study from Pakistan, where 49% of participants expressed concerns about increased costs after the placement of PEG tubes (31). Additionally, analyses from the US healthcare system indicate that there are substantial direct expenses associated with PEG placement, with wide variability due to complications and ongoing care needs (46). Although these findings suggest that the perceived financial impact of PEG may depend on the structure and coverage of the healthcare system, more detailed cost-effectiveness analyses specific to ALS are still needed.

The results of this study also indicate a positive effect of PEG on HRQoL measures, which may be unexpected in the context of terminal phases of such rapidly progressive neurodegenerative diseases (23). Notably, this effect was detected by applying the GIQLI, a scale specifically designed for assessing gastrointestinal-related HRQoL, on which PEG intervention was hypothetically expected to have a beneficial influence (32, 33). More specifically, the study revealed a significant improvement in the GIQLI gastrointestinal digestion subscale, which was maintained at follow-up, supporting the need for the application of appropriate outcome measures tailored to the specificity of the intervention applied. Interestingly, any significant worsening of the gastrointestinal defecation subscale was not observed, after PEG, which is notable since many patients may experience gastrointestinal complications because of enteral nutrition, such as diarrhea, nausea, cramps/bloating, and constipation. However, the improvement in the GIQLI was paralleled by a deterioration in HRQoL measures related to physical functioning, as consistently shown by the SF-36 PCS summary score and the GIQLI physical dimension subscale (23).

Although no previous studies are directly comparable to this study, the findings offer preliminary evidence of a positive perception of PEG among the ALS patients recruited in this study. Furthermore, PEG placement may contribute to improving HRQoL aspects associated with feeding and digestion, aligning with the intended therapeutic goals of the procedure. Nonetheless, this study has several limitations, including a relatively small sample size, the absence of matched control groups, and incomplete data regarding perspectives and HRQoL of all PEG recipients, which may introduce bias. Indeed, a major limitation of this study is that it failed to systematically collect quality-of-life data and patient perspectives from the entire cohort, and that control data were lacking. These issues may have introduced a relevant selection bias. Specifically, ALS patients who accept PEG placement differ inherently from those who decline it or are not eligible for the procedure, both clinically and functionally. This creates distinct clinical and psychological subgroups (5, 6, 22, 40). While this heterogeneity limits the validity of direct comparisons between the two groups in survival studies, it may also have introduced a potentially significant selection bias that could have influenced our findings and may limit the generalizability of the results to the broader ALS population. To mitigate this potential bias, sensitivity analyses and inverse probability weighting were conducted, which were consistent with our primary findings. Additionally, E-value analysis enabled us to quantify the degree of unmeasured confounding required to nullify these findings. An additional limitation of this study is the extended period over which data were collected, which may introduce bias due to evolving PEG techniques and changing clinical standards. Although each patient and their relatives received comprehensive information regarding potential risks, benefits, and the optimal timing for PEG placement, the absence of measures to control for expectation bias was a limitation (47). This bias may partly explain the discrepancy between satisfaction questionnaire responses, which indicate that patients believe PEG prolongs survival, and the current evidence, which does not conclusively support this assumption (6, 22). Recent qualitative research has shown that patients with ALS and their caregivers weigh clinical indications against personal values and expectations surrounding PEG placement (41, 45). These expectations, whether optimistic or apprehensive, can influence an individual’s perceived agency and satisfaction with their decision, independent of actual clinical outcomes. Moreover, standardized post-PEG measurements for weight stabilization or gain were not consistently available for our cohort, representing an additional significant limitation, since these nutritional outcomes may indeed influence patient and caregiver perceptions of the intervention. Additionally, in this study, we used the GIQLI scale, which is a general gastrointestinal HRQoL measure rather than a dysphagia-specific instrument (32). Although the scale was originally developed particularly for patients with esophageal or gastroesophageal symptoms and dysphagia, we used it to capture potential changes in HRQoL related to dysphagia and gastrointestinal symptoms, with the aim to better understand the complex deterioration in HRQoL associated with the multi-functional ALS disease progression. However, oropharyngeal dysphagia is the predominant dysphagia phenotype in ALS, affecting a large proportion of patients over the course of the disease and serving as the main indication for PEG placement (1, 2, 6). This feature of ALS-related dysphagia highlights a mismatch between the GIQLI’s original target construct and the clinical presentation typical of ALS, representing an additional limitation of this study. These constraints are partly attributable to the observational design of the study and the inherent complexity of managing patients in the advanced stages of ALS. Despite these limitations, our study contributes to the understanding of real-world PEG outcomes in ALS, a context in which randomized trials may be ethically unfeasible, making observational data the best available evidence.

The cornerstone of ALS management still relies on symptom control and improvement in quality of life. PEG positioning still remains a complex decision for ALS patients. While there is consensus that the insertion of a feeding tube may exert a positive effect on nutritional status and survival when timely discussed and planned (9), little is still known about patients’ perspectives on this intervention (4, 5). Although further research is needed to confirm our findings, this study indicates that patients have a positive perspective on PEG positioning, which may also have beneficial effects on their HRQoL.

## Data Availability

The raw data supporting the conclusions of this article will be made available by the authors, without undue reservation.
