# Supplementary material for: Perspective and quality of life in amyotrophic lateral sclerosis patients undergoing percutaneous endoscopic gastrostomy
Source: Front Nutr. 2026 Jun 19;13:1756642. doi: 10.3389/fnut.2026.1756642 (PMC13330603; doi:10.3389/fnut.2026.1756642)
Supplement: Supplementary file 1 [file Data_Sheet_1.PDF]

frontiers

Online supplementary material

Perspective and Quality of life in Amyotrophic Lateral Sclerosis patients undergoing percutaneous endoscopic gastrostomy

Nilo Riva,[1,2#] Enrica Finotto,[1] Paride Schito,[1,2] Donzelli Giorgia,[1] Tommaso Russo,[1,2] Teuta Domi,[2] Laura Pozzi,[2] Andrea Tettamanti,[3] Elisa Riboldi,[3] Ignazio Diego Lopez,[1] Angelo Quattrini,[2] George Cremona,[4] Mauro Comola,[1] Filippi Massimo[1,5]

Perspective and Quality of life in Amyotrophic Lateral Sclerosis patients undergoing percutaneous endoscopic gastrostomy

Nilo Riva,[1,2,#] Enrica Finotto,[1] Paride Schito,[1,2] Donzelli Giorgia,[1] Tommaso Russo,[1,2] Teuta Domi,[2] Laura Pozzi,[2] Andrea Tettamanti,[3] Elisa Riboldi,[3] Ignazio Diego Lopez,[1] Angelo Quattrini,[2] George Cremona,[4] Mauro Comola,[1] Filippi Massimo[1,5]

1 Neurology and Neuro-rehabilitation Unit, Division of Neuroscience, IRCCS San Raffaele, Milan, Italy

2 Experimental Neuropathology Unit, Division of Neuroscience, Institute of Experimental Neurology (INSPE), San Raffaele Scientific Institute, Milan, Italy.

3 Department of Rehabilitation and Functional Recovery, IRCCS San Raffaele Scientific Institute, Milan, Italy.

4 Unit of Respiratory Medicine, IRCCS San Raffaele Scientific Institute, Milan, Italy.

5 Vita-Salute San Raffaele University, Milan, Italy.

# Present address: IRCCS Istituto Neurologico Carlo Besta, Milan, Italy

Correspondence: nilomnd@gmail.com; filippi.massimo@hsr.it

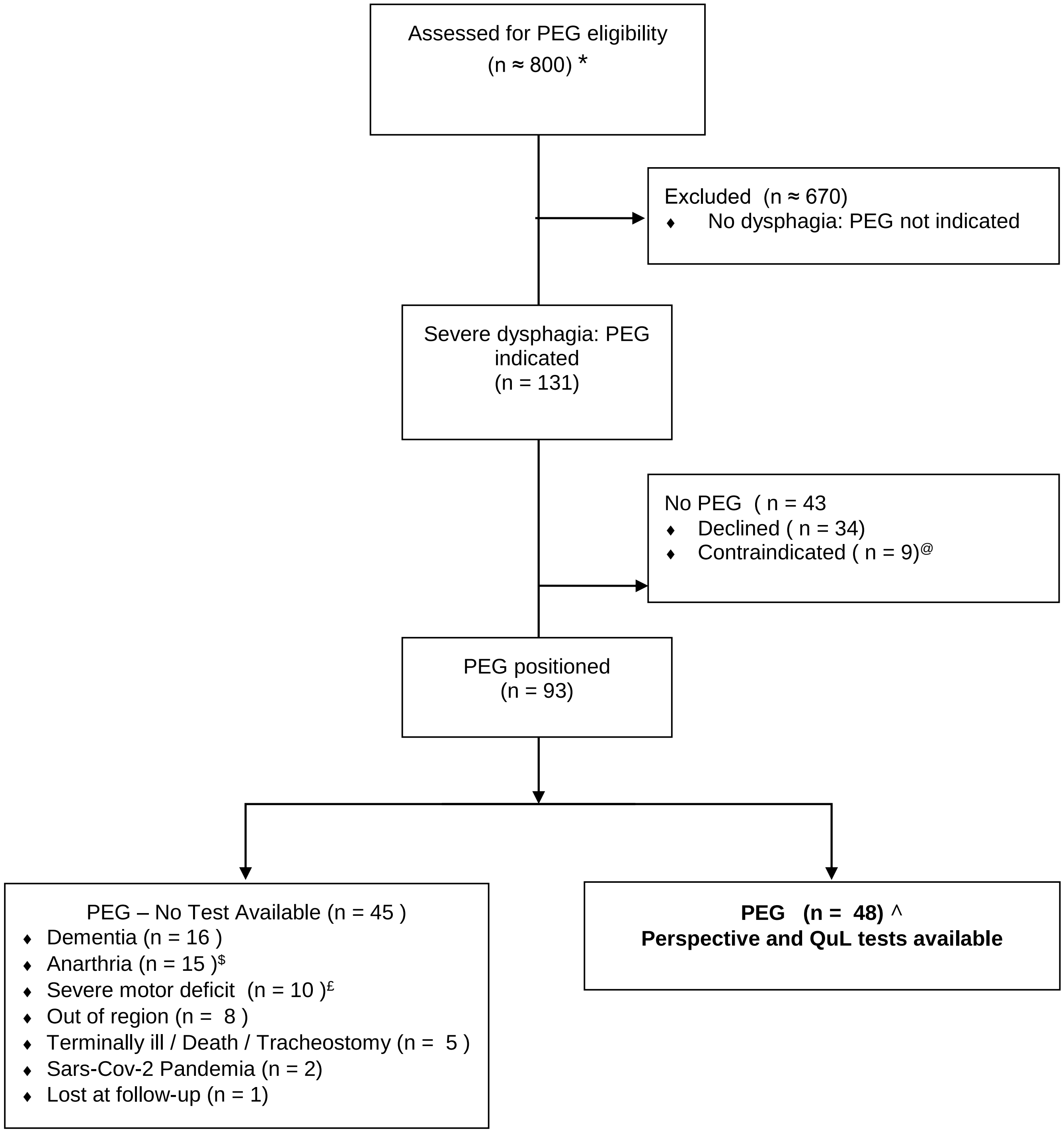

Figure S1: Patient Selection Flow-Chart.

Figure Legend: *: Admitted for Neuro-rehabilitation (2007-2020); [@]: high-risk/low-benefit, terminal stage, comorbidities; [$]: ALS-FRS-R item 1 = 0; [£]: ALS-FRS-R < 15; ^ Study population recruited in the Perspective and QoL study.

Table S1: Descriptive statistics comparing patients who did not underwent PEG versus PEG-recipients.

	GROUP	
	No PEG ***n: 42#***	***PEG*** ***n: 93***
**Age at onset (years)**	67.5 (8.4)	65.4 (10.2)
**Gender (M:F)**	23: 19 (54.8%: 45.2%)	35: 58 (37.6%: 62.4%)
**Diagnostic Delay(months)**	18.4 (25.9)	15.1 (24.1)
**Site of onset (bulbar:spinal)**	12: 30 (28.6%: 71.4%)	45: 48 (48.4%: 51.6%)*
**Disease duration at PEG (months)**	27.9 (28.6)	32.7 (36.5)
**El Escorial (definite: probable: probable lab-supported : possible)**	15: 14: 4: 9 (35.7%: 33.3%: 9.5%: 21.4%)	48: 20: 9: 16 (51.6%: 21.5%: 9.7%: 17.2%)
**ALS-FRS-R Total**	23.8 (9.7) (n:37)	24.5 (8.9) (n: 91)
**ALSFRS-R bulbar score**	6.8 (3.2) (n:37)	5.2 (2.9)* (n: 91)
**ALS-FRS-R Respiratory score**	7.7 (3.3) (n:37)	9.6 (2.5)*(n: 91)
**ALSFRS-R progression rate**	1.6 (1.3) (n:37)	1.2 (0.9)* (n: 91)
**MRC sum score**	78.8 (25.8) (n:37)	79.1 (30.3) (n: 88)
**UMN score**	8.7 (4.7)	10.4 (4.4)*
**Dementia (yes:no)**	5: 37 (11.9%: 88.1%)	23: 70 (24.7%: 75.3%)
**BMI (kg/m2)**	21.9 (3.4) (n:38)	21.2 (3.6) (n: 91)
**Weight loss in previous 5 months (kg) (%)**	6.0 (6.7) (n:30) 9.2 (10.2)	5.7 (5.5) (n:49) 8.7 (8.1)
**BMR (H-B) (cal/day)**	1231.0 (214.1) (n:38)	1207.0 (189.9) (n:92)
**Metabolism (normal: hypermetabolic: hypometabolic)**	8: 7: 7 (36.4%: 31.8%: 31.8%) (n:22)	30: 14: 24 (44.1%: 20.6%: 35.3%) (n:68)
**FVC %**	51.5 (25.1) (n:30)	59.1 (22.3) (n:67)
**NIV (yes: no)**	28: 14 (66.7%: 33.3%)	45: 48 (48.4%: 51.6%)*
**pO2**	79.6 (14.8) (n:37)	81.6 (17.2) (n:87)
**pCO2**	47.0 (9.2) (n:37)	43.0 (7.4)* (n:87)
**Genetic Tests (negative: not tested positive: specify**	31: 9 2: *C9orf72* RE, *TBK1* p.Lys291Glu	58: 33 2: *C9orf72* RE; *TARBP43* p.Ala382Thr

Values are presented as number of subjects (%) and mean values (SD); # Data not available for the all study group are specified in brackets. BMI: body mass index; BMR: basic metabolic rate; H-B: Herris Benedict. # Hyper-hypometabolism defined as: measured resting energy expenditure (MREE) at indirect calorimetry / HB-predicted REE > 110% or < 110%, respectively. FVC: forced vital capacity; NIV: non-invasive ventilation. Basal metabolic rate (BMR) was calculated for all patients with the Harris-Benedict (HB) predictive equation, while Resting energy expenditure (REE) was measured with indirect calorimetry (IC); patients were defined as hypermetabolic or hypometabolic when REE was > or < 110% of

predicted BMR, respectively (1). Genetic screening for major ALS-related genes was performed, as described (2). RE: repeat expansion; *: p< 0.05

Table S2: Descriptive statistics comparing patients who did not underwent PEG versus PEG-recipients, subdivided in patients for whom perspective and QoL tests were not available and patients from whom tests were obtained (Study population)

	Group		
	No PEG (A) n: 42	***PEG - No Test (B)*** n: 45	***PEG - Test (C)*** n: 48
**Age at onset (years)**	67.5 (8.4)	65.7 (9.3)	65.0 (11.1)
**Gender (M:F)**	23:19 (54.8%:45.2%)	17:28 (37.8%:62.2%)	18:30 (37.5%:62.5%)
**Diagnostic Delay(months)**	18.4 (25.9)	13.7 (20.0)	16.5 (27.5)
**Site of onset (Bulbar / spinal)**	12:30 (28.6%:71.4%)	22:23 (48.9%:51.1%)	23:25 (47.9%:52.1%)
**Disease duration at PEG (months)**	27.9 (28.6)	28.7 (23.8)	36.4 (45.3)
**El Escorial (definite: probable: probable lab-supported : possible)**	15 : 14 : 4 : 9 (35.7% : 33.3% : 9.5% : 21.4%)	24 : 6 : 5 : 10 (53.3%: 13.3% : 11.1% : 22.2%)	24 : 14 : 4 : 6 (50.0% : 29.2% - 8.3% - 12.5%)
**ALS-FRS-R Total**	23.8 (9.7) (n:37)	22.9 (8.8) (n:43)	25.9 (8.9)
**ALSFRS-R bulbar score**	6.8 (3.2) (n:37) ***B***	4.7 (2.9) (n:43)	5.7 (2.8)
**ALS-FRS-R Respiratory score**	7.7 (3.3) (n:37)	9.6 (2.6) (n:43) ***A***	9.6 (2.4) ***A***
**ALSFRS-R progression rate**	1.6 (1.3) (n:37)	1.2 (0.9) (n:43)	1.1 (0.9)
**MRC sum score**	78.8 (25.8) (n:37)	74.3 (31.4) (n:40)	83.1 (29.1)
**UMN score**	8.7 (4.7)	10.4 (4.3)	10.4 (4.6)
**Dementia (yes:no)**	5 : 37 (11.9% : 88.1%)	16 : 29 (35.6% : 64.4%) ***A C***	7 : 41 (14.6% : 85.4%)
**BMI (kg/m2)**	21.9 (3.4) (n:38)	21.0 (3.2) n: 43)	21.5 (4.0)
**Weight loss in the previous 3-6 months (kg)** **(%)**	6.0 (6.7) (n:30) 9.2 (10.2)	5.9 (5.8) (n:28) 8.9 (8.6)	5.5 (5.3) (n:21) 8.5 (7.7)
**BMR (H-B) (cal/day)**	1231.0 (214.1) (n:38)	1207.1 (163.7) (n:44)	1245.1 (211.3)
**Metabolism (normal : hypermetabolic : hypometabolic)#**	8 : 7 : 7 (36.4% : 31.8% : 31.8%) (n:22)	13 : 6 : 10 (44.8% : 20.7% : 34.5 %) (n:29)	17 : 8 : 14 (43.6% : 20.5% : 35.9%) (n:39)
**FVC %**	51.5 (25.1) (n:30)	50.0 (18.7) (n:28)	65.8 (22.4) (n:39) ***A B***
**NIV (yes:no)**	28 : 14 (66.7% : 33.3%)	22 : 23 (48.9%: 51.1%)	23:25 (47.9% : 52.1%)
**pO2**	79.6 (14.8) (n:37)	79.2 (17.7) (n:42)	83.9 (16.5) (n:45)
**pCO2**	47.0 (9.2) (n:37) ***C***	43.2 (9.6) (n:42)	42.8 (4.5) (n:45)

Values are presented as number of subjects (%) and mean values (SD); # Data not available for the all study group are specified in brackets. BMI: body mass index; BMR: basic metabolic rate; H-B: Herris Benedict. # Hyper-hypometabolism defined as: measured resting energy expenditure (MREE) at indirect calorimetry / HB-predicted REE > 110% or < 110%, respectively. FVC: forced vital capacity; NIV: non-invasive ventilation. Results are based on One-way ANOVA followed by Bonferroni post-Hoc test. For each significant pair, the key occurs in the category with the larger mean. Significance level for capital letters (A, B, C): $p< 0.05$

Table S3: Factors predicting survival in ALS patients from disease onset and from PEG recommendation (Univariate analysis)

Variable	From disease onset			From PEG recommendation		
	HR	*95% CI*	*p*	*HR*	*95% CI*	*p*
**PEG (yes:no)**	0.692	0.473-1.012	0.058	0.576	0.392-0.847	***0.005***
**Age at onset (years)**	1.031	1.012-1.051	***0.002***	1.018	0.999-1.037	0.61
**Gender (M:F)**	0.903	0.63-1.293	0.575	1.44	0.997-2.069	0.52
**Diagnostic Delay(months)**	0.971	0.958-0.985	***0.0001***	0.995	0.987-1.003	0.208
**Site of onset (bulbar / spinal)**	1.440	0.999-2.076	***0.051***	0.796	0.554-1.141	0.214
**Disease duration at PEG (months)**	0.965	0.956-0.976	***0.0001***	0.996	0.991-1.001	0.154
**El Escorial (Definite vs others)**	1.025	0.717-1.464	0.893	0.940	0.656-1.346	0.734
**ALS-FRS-R Total (n: 128)**	1.017	0.995-1.041	0.131	0.983	0.964-1.003	0.095
**ALSFRS-R bulbar score**	0.997	0.918-1.1041	0.471	0.1014	0.954-1-078	0.652
**ALS-FRS-R Respiratory score**	0.982	0.919-1.050	0.600	0.941	0.884-1.002	0.056
**ALSFRS-R progression rate**	2.356	1.957-2.836	***0.0001***	1.231	1.028-1.474	***0.024***
**MRC Sum score (n:125)**	1.004	0.998-1.010	0.195	0.996	0.990-1.002	0.191
**UMN score**	1.016	0.997-1.056	0.425	0.974	0.934-1.015	0.216
**Dementia (yes:no)**	1.305	0.844-2.016	0.231	1.136	0.735-1.756	0.565
**Riluzole (yes:no)**	0.702	0.433-1.136	0.149	0.528	0.323-0.864	***0.011***
**BMI (kg/m2)**	1.014	0.963-1.069	0.593	1.004	0.953-1.058	0.874
**BMI <18.5 kg/m2**	1.139	0.715-1.815	0.593	0.996	0.611-1.624	0.987
**Weight loss in the previous 3-6 months (n:89) (kg)**	1.068	1.027-1.110	***0.001***	1.035	0.997-1.073	0.068
**(%)**	1.041	1.014-1.068	***0.002***	1.030	1.003-1.057	***0.027***
**Weight loss > 10% (n: 89)**	1.770	1.107-2.831	***0.017***	1.458	0.921-2.307	0.107
**BMR (H-B) (cal/day) (n: 130)**	0.999	0.999-1	0. 064	0.999	0.998-1.000	0.053
**Hypometabolic (yes:no) (n: 90) #**	0.669	0.418-1.071	0.094	0.667	0.418-1.063	0.088
**FVC (%) at PEG (n: 97)**	0.994	0.985-1.003	0.216	0.987	0.978-0.997	***0.008***
**NIMV at PEG**	0.798	0.555-1.146	0,222	1.445	1.006-2.077	***0.047***
**pO2 (n:124)**	0.998	0.982-1.005	0.267	0.987	0.975-0.999	***0.041***
**pC02 (n:124)**	1.026	1.003-1.050	***0.025***	1.045	0.020-1.071	***0.0001***

Data not available for the all study group are specified in brackets. BMI: body mass index; BMR: basic metabolic rate; H-B: Herris Benedict. # Hyper-hypometabolism defined as: measured resting energy expenditure (MREE) at indirect calorimetry / HB-predicted REE > 110% or < 110%, respectively. FVC: forced vital capacity; NIV: non-invasive ventilation.

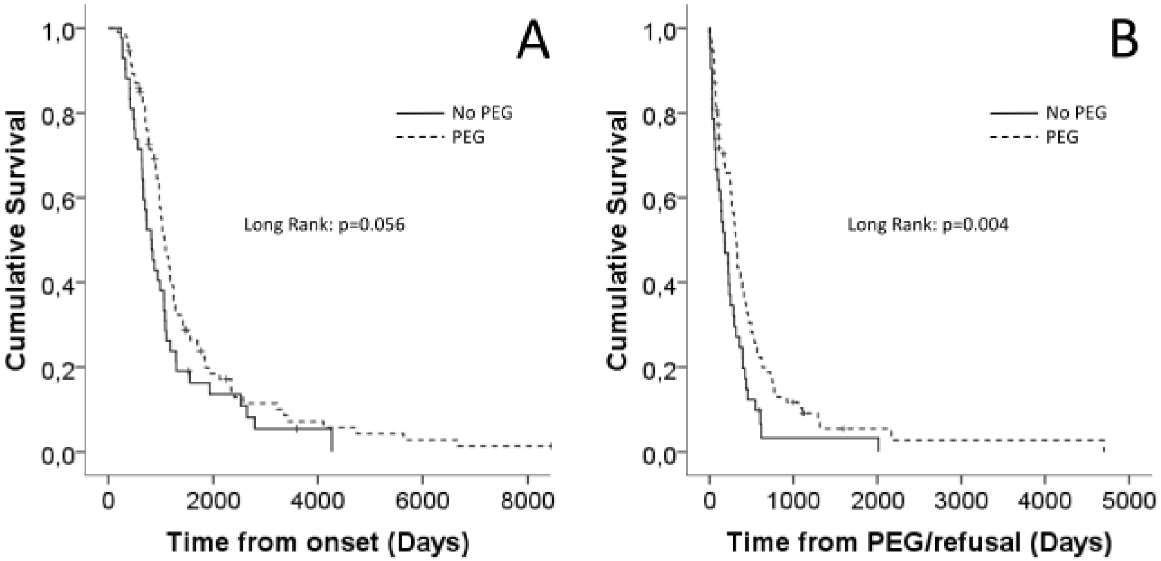

	PEG	No_PEG
Median	1079	821
SE	70.3	98.3

	PEG	No_PEG
Median	313	174
SE	21.4	51.7

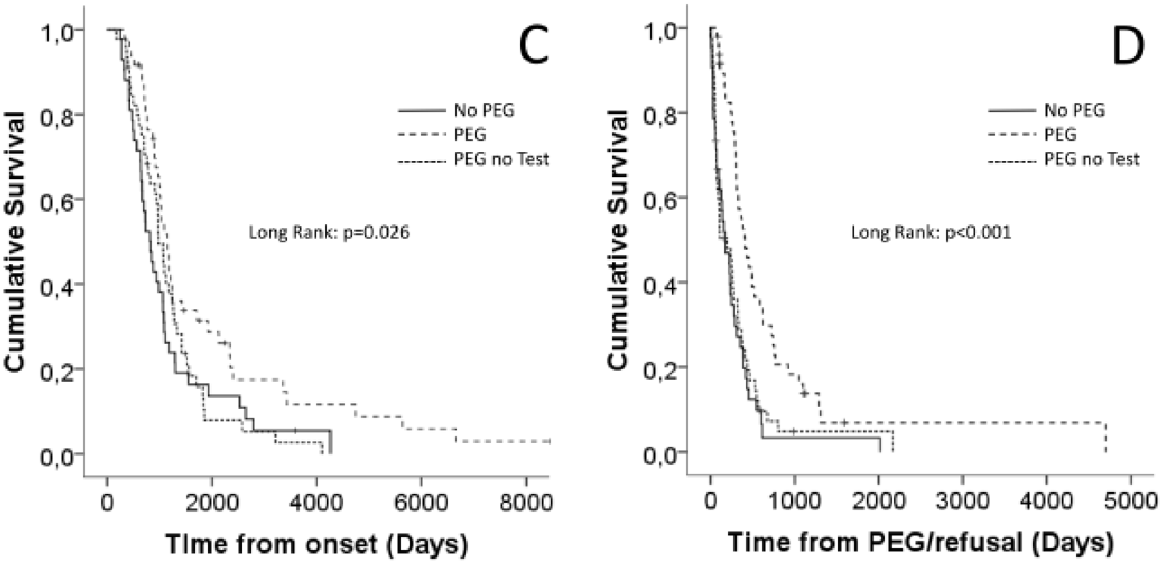

	PEG	PEG_No_Test	No_PEG
Median	409	198	174
SE	69.7	92.1	51.7

	PEG	PEG_No_Test	No_PEG
Median	1151	980	821
SE	85.5	68.5	98.3

Figure S2: Survival Analysis

Figure Legend: A and B: Kaplan-Mayer analysis comparing PEG and non-PEG recipients (A and B) and PEG patients for whom QoL tests were available, not available and non-PEG recipients (C and D). A and C: Overall survival: from symptomatic onset to death / tracheostomy; B and D: from PEG insertion / refusal to death / tracheostomy. p: Long-Rank (Mantel-Cox) test.

Table S4: Multivariate COX regression analysis for predicting survival

From Disease onset				
			95,0% CI for Exp(B)	
	p	***Exp(B)***	***Lower***	***Upper***
**PEG (yes:no)**	0.884	1.045	0.580	1.882
**ALSFRS-R progression rate**	***0.0001***	2.204	1.549	3.316
**Weight loss (previous 5 months; %)**	***0.040***	1.030	1.001	1.059
**Disease duration at PEG (months)**	***0.0001***	0.947	0.926	0.969
**pCO2**	***0.001***	1.055	1.024	1.087
Variables not included in the equation: Age at onset, diagnostic delay, Site of onset (bulbar:spinal)				
**From PEG recommendation**				
			95,0% CI for Exp(B)	
	p	***Exp(B)***	***Lower***	***Upper***
**PEG (yes:no)**	0.631	0.873	0.502	1,519
**pCO2**	***0.002***	1.047	1.018	1.078
**ALSFRS-R progression rate**	***0.004***	1.424	1.119	1.812
Variables not included in the equation: Weight loss (previous 3-6 months; %); Riluzole use, NIMV use				

Supplementary Methods: Sensitivity Analysis:

To mitigate potential selection bias related to the limited sample size, we undertook a series of sensitivity analyses within a causal inference framework. (n=48 of 131 PEG-considered patients with available perspective HRQoL data) we first conducted sensitivity analyses, including multiple imputation (MI) using baseline predictors to estimate missing values (Table S1), alongside complete case analysis and last observation carried forward (LOCF) approach (Table S6). Missing data were addressed using multiple imputation under a missing at random assumption conditional on observed baseline predictors, alongside a last observation carried forward approach as a deterministic comparator. To account for potential selection into response, we applied inverse probability weighting (IPW), estimating stabilized weights from a logistic model for response conditional on key clinical variables, including forced vital capacity, disease progression rate, disease duration at PEG placement, and ALSFRS-R score, using the full PEG cohort (n=88). To assess the stability of effect estimates, confidence intervals for changes in patient satisfaction and health-related quality of life were obtained using non-parametric bootstrapping with 1000 resamples. Finally, we calculated E-values to quantify the minimum strength of association that an unmeasured confounder would need to nullify findings.

Notably, both MI and LOCF approaches consistently confirmed a significant improvement of the GIQLI Gastrointestinal Digestion Subscale, despite a deterioration over time of the PCS SF-36 subscale. GIQLI Gastrointestinal Digestion Subscale improvement was associated in MI analysis with an amelioration at discharge of both the Mental Well-Being Subscale and the Total GIQLI scores, the letter confirmed at LOCF analysis. Additionally, MI analysis confirmed a significant deterioration over time of the Physical Dimension GIQLI subscale.

Table S5. Sensitivity Analysis - Multiple Imputation: values at baseline, discharge and at FU (Friedman Test)

Parameters	T0 - Baseline (pre-PEG)	T1 – Discharge	T2 – 1 month FU	*P*	*CI 95%*
SF-36					
Physical component summary (PCS)	30.36 (8.81)	27.95 (8.85)	27.98 (4.72)	**>0.0001**	*>0.0001 - 0.0005*
Mental component summary (MCS)	36.38 (11.66)	37.84 (11.48)	36.36 (7.26)	0.445	*0.442 - 0.468*
GIQLI					
Physical Well-Being Subscale	15.67 (6.36)	15.80 (5.80)	13.39 (10.111)	0.089	*0.082 - 0.097*
Physical Dimension	8.16 (5.59)	8.41 (5.31)	6.76 (4.84)	**0.040**	*0.035 - 0.045*
Mental Well-Being Subscale	8.54 (4.16)	9.59 (4.07)	8.61 (4.88)	**0.042**	*0.037 - 0.048*
Gastrointestinal Digestion Subscale	27.01 (5.68)	29.94 (5.50)	29.15 (8.31)	**>0.0001**	*>0.0001 - 0.0005*
Gastrointestinal Defecation Subscale	17.77 (3.66)	18.31 (4.41)	16.75 (5.13)	0.187	*0.177 - 0.197*
Total Subscales	78.71 (17.61)	83.30 (15.91)	77.89 (19.12)	**0.0002**	*>0.0001 - 0.001*

Values are presented as number of subjects (%) and mean values (SD); Sample size: SF-36: N: 93; GIQLI: N: 93 Friedman test. Post-hoc analysis shows significant results from discharge to follow-up (FU). Multiple imputation (N:5) analysis was performed, using baseline predictors (disease duration, Age, El-Escorial category, site of onset, FVC, ALSFRS-R scores, disease progression rate, MRC Sum scores, UMN score, Riluzole assumption and dementia status) to derive outcome measures; impudent data were subsequently pooled into a full dataset

Table S6. Senstivity Analysis – Last Observation Carry Forward (LOCF): values at baseline, discharge and at FU (Friedman Test)

SF-36					
**Parameters**	T0 - Baseline (pre-PEG)	T1 – Discharge	T2 – 1 month FU	*p*	*(95% CI)*
Physical component summary (PCS)	31.00 (9.70)	29.24(7.88)	28.58 (7.93)	**0.007**	0.005 - 0.009
Mental component summary (MCS)	35.26 (12.58)	37.15 (12.20)	36.28 (11.49)	0.388	0.375 -0.401
GIQLI					
Physical Well-Being Subscale	15.69 (7.08)	15.34 (6.94)	14.19 (6.56)	0.572	0.559 -0.585
Physical Dimension	8.31 (6.31)	7.91 (5.59)	7.07 (4.84)	0.247	0.235 -0.058
Mental Well-Being Subscale	8.62 (4.73)	9.26 (4.69)	8.85 (4.47)	0.289	0.277 -0.300
Gastrointestinal Digestion Subscale	27.15 (6.51)	29.53 (6.52)	29.37 (7.03)	**>0.0001**	>0.0001 - 0.0005
Gastrointestinal Defecation Subscale	17.91 (4.09)	18.25 (3.75)	17.44 (4.19)	0.170	0.160 -0.179
Total Subscales	79.25 (20.33)	82.01 (19.72)	79.16 (20.56)	**0.018**	0.014 - 0.021

Values are presented as number of subjects (%) and mean values (SD); Sample size: SF-36: N: 65; GIQLI: N: 68 Friedman test. Post-hoc analysis shows significant results from discharge to follow-up (FU).

Inverse probability weighting (IPW) analysis:

Table S7. Patients / Caregivers perspective regarding PEG-tube placement before and after IPW.

Patients								
	Unweighted (Before IPW)				***Weighted (After IPW)***			
**Question**	**Yes**	**No /Indecisive**	**|STD|**	***p***	**Yes**	**No / Indecisive**	**|STD|**	***P***
**Would you like to have PEG tube again?**	37 (77.1%)	11 (22.9%)	1.289	0.000	47 (74.6%)	16 (25.4%)	1.130	0.000
**Ease in feeding**	45 (93.8%)	3 (6.2%)	3.615	0.000	62 (98.4%)	1 (1.6%)	7.747	0.000
**Cosmetically acceptable**	26 (54.2%)	22 (35.8%)	0.167	0.004	37 (58.7%)	26 (41.3%)	0.355	0.000
**PEG tube increases survival**	45 (93.8%)	3 (6.2%)	3.615	0.000	56 (88.9%)	7 (11.1%)	2.475	0.000
**Positive effect on overall well-being**	40 (83.3%)	8 (16.7%)	1.789	0.000	49 (77.8%)	14 (22.2%)	1.336	0.000
**Increase time for feeding**	30 (62.5%)	18 (37.5%)	0.516	0.000	42 (66.7%)	21 (33.3%)	0.707	0.000
**Dependent on the others for feeding**	35 (72.9%)	13 (27.1%)	1.031	0.000	48 (76.2%)	15 (23.8%)	1.230	0.000
**Increase in the cost of care**	29 (39.6%)	29 (60.4%)	0.000	0.022	30 (47.6%)	33 (52.4%)	0.095	0.000
**Caregivers (N: 60)**								
**Would you like to have PEG tube again?**	40 (88.9%)	5 (11.1%)	2.475	0.000	52 (86.7%)	8 (13.3%)	2.157	0.000
**Ease in feeding**	45 (100%)	0	-	0.000	60 (100%)	0	-	0.000
**Cosmetically acceptable**	31 (68.8%)	14 (31.2%)	0.816	0.000	42 (70.0%)	18 (30.0%)	0.873	0.000
**PEG tube increases survival**	43 (95.5%)	2 (4.5%)	4.421	0.000	57 (95.5%)	3 (5.0%)	4.129	0.000
**Positive effect on overall well-being**	37 (82.2%)	8 (17.8%)	1.686	0.000	49 (81.7%)	11 (18.3%)	1.637	0.000
**Increase time for feeding**	31 (68%)	14 (31.0%)	0.816	0.000	44 (73.3%)	16 (26.6%)	1.055	0.000
**Dependent on the others for feeding**	29 (64.4%)	16 (35.6%)	0.604	0.000	43 (71.7%)	17 (28.3%)	0.962	0.000
**Increase in the cost of care**	12 (26.7%)	33 (73.3%)	1.055	0.005	18 (30.0%)	42 (70.0%)	0.873	0.000

Values are shown as the number of subjects (%). To compare categorical variables. a two-tailed p value Fisher exact test of k-proportions was applied. STD: standardized mean difference

Table S8. values at baseline. discharge and at FU (Friedman Test) before and after IPW.

SF-36														
	Unweighted (Before IPTW)							***Weighted (After IPTW)***						
**Parameters**	**T0**	**T1**	**T2**	**|STD| T1-T0**	**|STD| T2-T0**	**|STD| T3-T2**	***p***	**T0**	**T1**	**T2**	**|STD| T1-T0**	**|STD| T2-T0**	**|STD| T3-T2**	***p***
PCS	33.28 (10.86)	30.37 (8.27)	28.61 (8.70)	0.32	0.57**	0.17	***0.034***	31.24 (10.47)	30.18 (8.51)	27.80 (7.86)	0.15	0.50	0.21	0.131
MCS	35.21 (12.55)	38.18 (13.05)	36.83 (12.70)	0.25	0.10	0.10	0.605	35.18 (11.32)	37.24 (12.52)	37.00 (11.89)	0.13	0.02	0.02	0.352
GIQLI														
Physical Well-Being Subscale	17.17 (6.88)	16.31 (6.43)	13.83 (5.32)	0.14	0.46	0.36	0.404	16.19 (6.13)	16.14 (5.64)	14.17 (5.24)	0.03	0.34	0.39	0.511
Physical Dimension	10.48 (6.69)	9.17 (5.81)	7.10 (4.60)	0.19	0.44*	0.43*	***0.045***	9.95 (6.34)	9.48 (5.35)	7.38 (4.50)	0.09	0.43**	0.47**	***0.0001***
Mental Well-Being Subscale	8.97 (4.23)	8.83 (4.08)	8.97 (4.10)	0.07	0.17*	0.06	0.717	8.83 (4.05)	9.12 (4.21)	9.17 (3.90)	0.17	0.06	0.04	0.194
Gastrointestinal Digestion Subscale	28.38 (6.60)	30.97 (5.84)	30.21 (6.67)	0.60**	0.33	-0.14	**0.014**	28.76 (6.18)	31.52 (5.74)	30.24 (5.66)	0.61***	0.27	0.24	***0.001***
Gastrointestinal Defecation Subscale	18.72 (3.73)	18.97 (3.27)	17.28 (4.54)	1.40	0.38	0.44	0.177	18.12 (3.86)	18.62 (3.35)	17.00 (4.41)	1.52	0.32	0.49	0.088
Total	83.72 (17.07)	86.95 (17.02)	79.10 (19.25)	0.17	0.29	0.42	0.072	82.48 (16.29)	85.57 (16.21)	79.17 (18.53)	0.28*	0.24	0.56*	***0.000***

*Values are presented as number of subjects (%) and mean values (SD); Sample size (unweighted): Sample size: SF-36: N: 23; GIQLI: N: 29. T0: Baseline (Pre-PEG); T1: Discharge; T2: 1 month FU; PCS: physical component summary; MCS: mental component summary. Friedman test. Post-Hoc: *:p<0.05; **:p<0.005; ***:p<0.0005; STD: standardized mean difference for repeated measure*

Table S9. Subgroup Analysis: Patients / Caregivers perspective regarding PEG-tube placement: Spinal patients

**Patients (N: 25)**					
**Question**	**Yes**	**No**	**Indecisive**	**p-value**	**95% CI**
**Would you like to have PEG tube again?**	20 (80%)	4 (16%)	1 (4%)	0.000	0.000 - 0.0005
**Ease in feeding**	25 (100%)	0 (0%)	0 (0%)	0.000	0.000 - 0.0005
**Cosmetically acceptable**	14 (56%)	7 (28%)	4 (16%)	0.038	0.033 - 0.043
**PEG tube increases the survival**	24 (96%)	1 (4%)	0 (0%)	0.000	0.000 - 0.0005
**Positive effect on overall well-being**	19 (76%)	2 (8%)	4 (16%)	0.000	0.000 - 0.005
**Increase time consumption due to feeding**	14 (56%)	7 (28%)	4 (16%)	0.038	0.033 - 0.0043
**Dependent on the others for feeding**	17 (68%)	6 (24%)	2 (8%)	0.001	0.000 - 0.001
**Increase in the cost of care**	9 (36%)	13 (52%)	3 (12%)	0.046	0.041 -0.051
**Caregivers (N: 24)**					
**Question**	**Yes**	**No**	**Indecisive**	**p-value**	**95% CI**
**Would you like to have PEG tube again?**	22 (92%)	2 (8%)	0 (0%)	0.000	0.000 - 0.004
**Ease in feeding**	24 (100%)	0 (0%)	0 (0%)	0.000	0.000 - 0.0004
**Cosmetically acceptable**	15 (63%)	4 (17%)	5 (21%)	0.011	0.008 -0.013
**PEG tube increases the survival**	24 (100%)	0 (0%)	0 (0%)	0.000	0.000 - 0.0004
**Positive effect on overall well-being**	19 (79%)	1 (4%)	4 (17%)	0.000	0.000 - 0.004
**Increase time consumption due to feeding**	15 (63%)	5 (21%)	4 (17%)	0.011	0.008 -0.0013
**Dependent on the others for feeding**	14 (58%)	8 (33%)	2 (8%)	0.012	0.009 - 0.015
**Increase in the cost of care**	5 (21%)	15 (63%	4 (17%)	0.011	0.008 -0.013

Table S10. Subgroup Analysis: Patients / Caregivers perspective regarding PEG-tube placement: Bulbar patients

Patients (N: 23)					
**Question**	**Yes**	**No**	**Indecisive**	**p-value**	**95% CI**
**Would you like to have PEG tube again?**	17 (74%)	4 (17%)	2 (9%)	0.000	0.000 - 0.001
**Ease in feeding**	20 (87%)	1 (4%)	2 (9%)	0.000	0.000 - 0.000
**Cosmetically acceptable**	12 (52%)	7 (30%)	4 (17%)	0.136	0.127 - 0.145
**PEG tube increases the survival**	21 (91%)	0 (0%)	2 (9%)	0.000	0.000 - 0.000
**Positive effect on overall well-being**	21 (91%)	1 (4%)	1 (4%)	0.000	0.000 - 0.000
**Increase time consumption due to feeding**	16 (70%)	4 (17%)	3 (13%)	0.001	0.000 - 0.002
**Dependent on the others for feeding**	18 (78%)	5 (22%)	0 (0%)	0.009	0.006 - 0.0011
**Increase in the cost of care**	10 (43%)	9 (39%)	4 (17%)	0.275	0.264 -0.287
**Caregivers (N: 21)**					
**Question**	**Yes**	**No**	**Indecisive**	**p-value**	**95% CI**
**Would you like to have PEG tube again?**	18 (86%)	0 (0%)	3 (14%)	0.001	0.000 - 0.002
**Ease in feeding**	21 (100%)	0 (0%)	0 (0%)	0.000	0.000 - 0.001
**Cosmetically acceptable**	16 (76%)	2 (10%)	3 (14%)	0.000	0.000 - 0.001
**PEG tube increases the survival**	19 (90%)	0 (0%)	2 (10%)	0.000	0.000 - 0.001
**Positive effect on overall well-being**	18 (86%)	0 (0%)	3 (14%)	0.001	0.000 - 0.002
**Increase time consumption due to feeding**	16 (76%)	2 (10%)	3 (14%)	0.000	0.000 - 0.001
**Dependent on the others for feeding**	15 (71%)	5 (24%)	1 (5%)	0.001	0.000 - 0.001
**Increase in the cost of care**	7 (33%)	10 (48%)	4 (19%)	0.304	0.292 - 0.315

Values are shown as the number of subjects (%). To compare categorical variables. a two-tailed p value Fisher exact test of k-proportions was applied

Table S11. Subgroup Analysis: values at baseline. discharge and at FU (Friedman Test): Spinal Patients

SF-36					
**Parameters**	T0	T1	T2	*p*	*CI 95%*
PCS	28.09 (7.96)	27.88 (5.24)	25.69 (5.53)	0.620	0.608 – 0.633
MCS	35.01 (15.77)	38.34 (15.68)	38.03 (15.83)	0.167	0.157 – 0.176
GIQLI					
Physical Well-Being Subscale	15.67 (740)	14.73 (6.50)	12.47 (4.74)	0.746	0.734 – 0.757
Physical Dimension	9.20 (7.45)	8.67 (5.79)	6.00 (4.01)	0.039	0.034 – 0.044
Mental Well-Being Subscale	8.33 (4.93)	9.13 (4.68)	8.27 (4.21)	0.301	0.289 – 0.313
Gastrointestinal Digestion Subscale	28.73 (6.99)	30.60 (6.30)	30.53 (6.12)	0.145	0.136 – 0.154
Gastrointestinal Defecation Subscale	18.27 (3.65)	18.67 (4.11)	17.87 (4.12)	0.838	0.828 – 0.847
Total	81.00 (20.73)	82.87 (18.61)	78.00 (17.87)	0.293	0.281 – 0.304

Table S14. Subgroup Analysis: values at baseline. discharge and at FU (Friedman Test): Bulbar patients

SF-36					
**Parameters**	T0	T1	T2	*p*	*CI 95%*
PCS	28.09 (7.96)	27.88 (5.24)	25.69 (5.53)	0.006	0.004 – 0.008
MCS	35.39 (9.41)	38.04 (10.81)	35.73 (9.60)	0.753	0.742 – 0.764
GIQLI					
Physical Well-Being Subscale	18.79 (6.12)	18.00 (6.11)	15.29 (5.67)	0.456	0.444 – 0.469
Physical Dimension	11.86 (5.70)	9.71 (5.99)	8.29 (5.03)	0.262	0.251 – 0.274
Mental Well-Being Subscale	9.64 (3.36)	8.50 (3.45)	9.71 (3.98)	0.475	0.462 – 0.488
Gastrointestinal Digestion Subscale	28.00 (6.39)	31.36 (5.51)	29.86 (7.42)	0.108	0.100 – 0.116
Gastrointestinal Defecation Subscale	19.21 (3.88)	19.29 (2.12)	16.64 (5.03)	0.110	0.102 – 0.118
Total	86.64 (12.12)	87.00 (15.54)	80.29 (21.24)	0.223	0.212 – 0.234

T0: Baseline (Pre-PEG); T1: Discharge; T2: 1 month FU; PCS: physical component summary; MCS: mental component summary.

Table S12. Subgroup Analysis: Patients / Caregivers perspective regarding PEG-tube placement: NIMV: NO

Patients (N: 25)					
**Question**	**Yes**	**No**	**Indecisive**	**p-value**	**95% CI**
**Would you like to have PEG tube again?**	19 (76%)	4 (16%)	2 (8%)	0.000	0.000 – 0.000
**Ease in feeding**	24 (96%)	0 (0%)	1 (4%)	0.000	0.000 – 0.000
**Cosmetically acceptable**	14 (56%)	7 (28%)	4 (16%)	0.039	0.034 – 0.044
**PEG tube increases the survival**	23 (92%)	1 (4%)	1 (4%)	0.000	0.000 – 0.000
**Positive effect on overall well-being**	20 (80%)	2 (8%)	3 (12%)	0.000	0.000 – 0.000
**Increase time consumption due to feeding**	14 (56%)	6 (24%)	5 (20%)	0.062	0.056 – 0.068
**Dependent on the others for feeding**	18 (72%)	6 (24%)	1 (4%)	0.000	0.000 – 0.000
**Increase in the cost of care**	12 (48%)	12 (48%)	1 (4%)	0.007	0.000 – 0.000
**Caregivers (N: 24)**					
**Question**	**Yes**	**No**	**Indecisive**	**p-value**	**95% CI**
**Would you like to have PEG tube again?**	20 (83%)	0 (0%)	4 (17%)	0.001	0.000 – 0.002
**Ease in feeding**	24 (100%)	0 (0%)	0 (0%)	0.000	0.000 – 0.000
**Cosmetically acceptable**	18 (75%)	2 (8%)	4 (17%)	0.000	0.000 – 0.000
**PEG tube increases the survival**	22 (92%)	0 (0%)	2 (8%)	0.000	0.000 – 0.000
**Positive effect on overall well-being**	19 (79%)	1 (4%)	4 (17%)	0.000	0.000 – 0.000
**Increase time consumption due to feeding**	16 (67%)	5 (21%)	3 (13%)	0.002	0.001 – 0.003
**Dependent on the others for feeding**	18 (75%)	5 (21%)	1 (4%)	0.000	0.000 – 0.000
**Increase in the cost of care**	9 (38%)	12 (50%)	3 (13%)	0.079	0.072 – 0.085

Values are shown as the number of subjects (%). NIMV: Non-Invasive - Ventilation

Table S13. Subgroup Analysis: Patients / Caregivers perspective regarding PEG-tube placement: NIMV YES

Patients (N: 23)					
**Question**	**Yes**	**No**	**Indecisive**	**p-value**	**95% CI**
**Would you like to have PEG tube again?**	18 (78%)	4 (17%)	1 (4%)	0.000	0.000 – 0.000
**Ease in feeding**	21 (91%)	1 (4%)	0 (0%)	0.000	0.000 – 0.000
**Cosmetically acceptable**	12 (52%)	7 (30%)	4 (17%)	0.138	0.000 – 0.000
**PEG tube increases the survival**	22 (96%)	0 (0%)	1 (4%)	0.000	-0.129 – 0.147
**Positive effect on overall well-being**	20 (87%)	1 (4%)	2 (9%)	0.000	0.000 – 0.000
**Increase time consumption due to feeding**	16 (70%)	5 (22%)	2 (9%)	0.001	0.000 – 0.001
**Dependent on the others for feeding**	17 (74%)	5 (22%)	1 (4%)	0.000	0.000 – 0.000
**Increase in the cost of care**	7 (30%)	10 (43%)	6 (26%)	0.655	0.643 – 0.668
**Caregivers (N: 21)**					
**Question**	**Yes**	**No**	**Indecisive**	**p-value**	**95% CI**
**Would you like to have PEG tube again?**	20 (95%)	0 (0%)	1 (5%)	0.000	0.000 – 0.000
**Ease in feeding**	20 (95%)	0 (0%)	1 (5%)	0.000	0.000 – 0.000
**Cosmetically acceptable**	13 (62%)	4 (19%)	4 (19%)	0.025	0.021 – 0.029
**PEG tube increases the survival**	21 (100%)	0 (0%)	0 (0%)	0.000	0.000 – 0.000
**Positive effect on overall well-being**	18 (86%)	0 (0%)	3 (14%)	0.001	0.000 – 0.002
**2Increase time consumption due to feeding**	15 (71%)	2 (10%)	4 (19%)	0.001	0.000 – 0.001
**Dependent on the others for feeding**	11 (52%)	8 (38%)	2 (10%)	0.052	0.046 – 0.057
**Increase in the cost of care**	3 (14%)	13 (62%)	5 (24%)	0.021	0.018 – 0.025

Values are shown as the number of subjects (%). NIMV: Non-Invasive - Ventilation

Table S14. Subgroup Analysis: values at baseline. discharge and at FU (Friedman Test) NIMV NO

SF-36					
**Parameters**	T0	T1	T2	*p*	*CI 95%*
PCS	35.89 (12.31)	30.52 (9.38)	28.88 (10.15)	0.025	0.021 – 0.029
MCS	33.84 (7.36)	39.34 (11.96)	38.00 (9.21)	0.324	0.312 – 0.336
GIQLI					
Physical Well-Being Subscale	20.47 (6.12)	18.06 (6.088)	15.35 (5.20)	0.183	0.173 – 0.193
Physical Dimension	13.12 (6.97)	10.88 (6.35)	8.18 (4.65)	0.040	0.035 – 0.045
Mental Well-Being Subscale	9.53 (4.23)	9.18 (3.94)	9.53 (3.41)	0.788	0.777 – 0.798
Gastrointestinal Digestion Subscale	29.06 (7.05)	31.35 (6.20)	30.71 (6.687)	0.139	0.130 – 0.148
Gastrointestinal Defecation Subscale	19.12 (3.82)	19.00 (3.18)	17.41 (4.47)	0.557	0.544 – 0.569
Total	89.00 (18.18)	87.71 (17.62)	82.12 (17.89)	0.304	0.292 – 0.316

Table S15. Subgroup Analysis: values at baseline. discharge and at FU (Friedman Test) NIMV YES

SF-36					
**Parameters**	T0	T1	T2	*p*	*CI 95%*
PCS	29.88 (7.94)	30.17 (7.06)	28.25 (6.87)	0.711	0.700 – 0.723
MCS	36.99 (17.50)	36.55 (14.86)	35.32 (16.64)	0.973	0.969 – 0.977
GIQLI					
Physical Well-Being Subscale	12.50 (5.03)	13.83 (6.30)	11.67 (4.88)	0.276	0.264 – 0.287
Physical Dimension	6.75 (4.15)	6.75 (4.15)	6.75 (4.25)	0.514	0.501 – 0.527
Mental Well-Being Subscale	8.17 (4.28)	8.33 (4.39)	8.17 (4.97)	0.924	0.917 – 0.930
Gastrointestinal Digestion Subscale	27.42 (6.067)	30.42 (5.50)	29.50 (6.86)	0.070	0.064 – 0.077
Gastrointestinal Defecation Subscale	18.17 (3.68)	18.92 (3.52)	17.08 (4.83)	0.258	0.247 – 0.269
Total	76.25 (12.52)	80.83 (15.97)	74.83 (21.06)	0.243	0.232 – 0.254

T0: Baseline (Pre-PEG); T1: Discharge; T2: 1 month FU; PCS: physical component summary; MCS: mental component summary.

E-Value analysis:

Table S16. Patients / Caregivers perspective regarding PEG-tube placement

Patients			
**Question**	**Yes**	**No /Indecisive**	**E-Value**
**Would you like to have PEG tube again?**	37 (77.1%)	11 (22.9%)	4.40
**Ease in feeding**	45 (93.8%)	3 (6.2%)	1.00
**Cosmetically acceptable**	26 (54.2%)	22 (35.8%)	5.92
**PEG tube increases survival**	45 (93.8%)	3 (6.2%)	53.15
**Positive effect on overall well-being**	40 (83.3%)	8 (16.7%)	1.60
**Increase time for feeding**	30 (62.5%)	18 (37.5%)	53.15
**Dependent on the others for feeding**	35 (72.9%)	13 (27.1%)	9.66
**Increase in the cost of care**	29 (39.6%)	29 (60.4%)	2.58
**Care-Givers**			
**Would you like to have PEG tube again?**	40 (88.9%)	5 (11.1%)	18.50
**Ease in feeding**	45 (100%)	0	-
**Cosmetically acceptable**	31 (68.8%)	14 (31.2%)	3.62
**PEG tube increases survival**	43 (95.5%)	2 (4.5%)	111.26
**Positive effect on overall well-being**	37 (82.2%)	8 (17.8%)	8.74
**Increase time for feeding**	31 (68%)	14 (31.0%)	3.62
**Dependent on the others for feeding**	29 (64.4%)	16 (35.6%)	2.86
**Increase in the cost of care**	12 (26.7%)	33 (73.3%)	4.67

Values are shown as the number of subjects (%). To compare categorical variables.

Table S17. **values at baseline. discharge and at FU (Friedman Test)**

SF-36							
**Parameters**	**T0**	**T1**	**T2**	**E-Value T1-T0**	**E-Value T2-T0**	**E-Value T3-T2**	***p***
PCS	33.28 (10.86)	30.37 (8.27)	28.61 (8.70)	2.01	2.76**	1.61	***0.034***
MCS	35.21 (12.55)	38.18 (13.05)	36.83 (12.70)	0.25	0.10	-0.10	0.605
GIQLI							
Physical Well-Being Subscale	17.17 (6.88)	16.31 (6.43)	13.83 (5.32)	1.65	2.36	2.31	0.404
Physical Dimension	10.48 (6.69)	9.17 (5.81)	7.10 (4.60)	1.52	2.41*	2.13*	***0.045***
Mental Well-Being Subscale	8.97 (4.23)	8.83 (4.08)	8.97 (4.10)	1.33	1.62	1.29	0.717
Gastrointestinal Digestion Subscale	28.38 (6.60)	30.97 (5.84)	30.21 (6.67)	2.86**	2.04	1.52	**0.014**
Gastrointestinal Defecation Subscale	18.72 (3.73)	18.97 (3.27)	17.28 (4.54)	6.62	2.17	2.36	0.38
Total	83.72 (17.07)	86.95 (17.02)	79.10 (19.25)	1.60	1.93	2.29	0.072

*Values are presented as number of subjects (%) and mean values (SD); Sample size: SF-36: N: 23; GIQLI: N: 29. T0: Baseline (Pre-PEG); T1: Discharge; T2: 1 month FU; PCS: physical component summary; MCS: mental component summary. Friedman test. Post-Hoc: *:p<0.05; **:p<0.005.*

REFERENCES

1. Dev R, Hui D, Chisholm G, Delgado-Guay M, Dalal S, Del Fabbro E, et al. Hypermetabolism and symptom burden in advanced cancer patients evaluated in a cachexia clinic. J Cachexia Sarcopenia Muscle. 2015;6(1):95-8.
2. Riva N, Pozzi L, Russo T, Pipitone GB, Schito P, Domi T, et al. NEK1 Variants in a Cohort of Italian Patients With Amyotrophic Lateral Sclerosis. Frontiers in neuroscience. 2022;16:833051.
